# Participation of hepatic α/β-adrenoceptors and AT_1_ receptors in glucose release and portal hypertensive response induced by adrenaline or angiotensin II

**DOI:** 10.1590/1414-431X20187526

**Published:** 2018-11-14

**Authors:** L.J.T. de Araújo, M.R. Nagaoka, D.R. Borges, M. Kouyoumdjian

**Affiliations:** 1 Departamento de Bioquímica, Escola Paulista de Medicina, Universidade Federal de São Paulo, São Paulo, SP, Brasil; 2 Departamento de Biociências, Universidade Federal de São Paulo, Baixada Santista, SP, Brasil; 3 Departamento de Medicina, Escola Paulista de Medicina, Universidade Federal de São Paulo, São Paulo, SP, Brasil

**Keywords:** Liver perfusion, Angiotensin II, Adrenaline, AT_1_R, Adrenoceptors

## Abstract

It has been previously demonstrated that the hemodynamic effect induced by angiotensin II (AII) in the liver was completely abolished by losartan while glucose release was partially affected by losartan. Angiotensin II type 1 (AT_1_) and adrenergic (∝1- and β-) receptors (AR) belong to the G-proteins superfamily, which signaling promote glycogen breakdown and glucose release. Interactive relationship between AR and AT_1_-R was shown after blockade of these receptors with specific antagonists. The isolated perfused rat liver was used to study hemodynamic and metabolic responses induced by AII and adrenaline (Adr) in the presence of AT_1_ (losartan) and ∝1-AR and β-AR antagonists (prazosin and propranolol). All antagonists diminished the hemodynamic response induced by Adr. Losartan abolished hemodynamic response induced by AII, and AR antagonists had no effect when used alone. When combined, the antagonists caused a decrease in the hemodynamic response. The metabolic response induced by Adr was mainly mediated by ∝_1_-AR. A significant decrease in the hemodynamic response induced by Adr caused by losartan confirmed the participation of AT_1_-R. The metabolic response induced by AII was impaired by propranolol, indicating the participation of β-AR. When both ARs were blocked, the hemodynamic and metabolic responses were impaired in a cumulative effect. These results suggested that both ARs might be responsible for AII effects. This possible cross-talk between β-AR and AT_1_-R signaling in the hepatocytes has yet to be investigated and should be considered in the design of specific drugs.

## Introduction

The first observations of the renin-angiotensin system (RAS) and pressor effects in the kidney and its role in hypertension was made in 1898 by Tigerstedt and Bergman (1). In 1976, Borges and co-works (2) described, for the first time, the hepatic conversion of angiotensin I to angiotensin II (AII), followed by AII inactivation. Further studies showed that AII produces an increase in the hepatic portal pressure and metabolic responses. The hemodynamic effect was completely abolished by losartan (angiotensin II type 1 receptor (AT_1_-R)-dependent mechanism) while metabolic responses such as glucose release and O_2_ consumption were partially affected by losartan (AT_1_-R-independent mechanism) (3). AT_1_ and adrenergic (∝1- and β-) receptors (AR) belong to the G-proteins superfamily that promote, following signalization, an increase of intracellular calcium that culminates with the glycogen breakdown and glucose release. An interactive relationship between AR and AT_1-_R was found after blockade of these receptors with specific antagonists (4,5). Exposure to elevated catecholamines or AII results in homologous desensitization of both adrenergic and AT_1_-mediated vascular smooth contraction in the rat or rabbit aorta ([Bibr B06] [Bibr B07] 68). This desensitization mediated by G-protein coupled receptors may result from changes in receptors, G proteins, carriers, or the interaction among these component systems (9,10). Therefore, selective antagonism of AR may clarify a possible alternative site for AII interaction, leading to glucose release. The present work was designed to study the effects of AT_1_R and AR blockade on AII-induced hepatic glucose release and portal pressure.

## Material and Methods

### Animals

Adult male Wistar rats (*Rattus norvergicus albinus*) (270–320 g) obtained from Centro de Desenvolvimento de Modelos Animais para Medicina e Biologia (CEDEME) of the Universidade Federal de São Paulo (UNIFESP) were fed standard laboratory diet (Purina®, Brasil) and water *ad libitum*. The animal experimental procedure was carried out in accordance to the guidelines of the Ethics in Research Committee of UNIFESP (CEP 1456/09).

### Liver perfusion

Rat liver perfusion was performed as previously described (11). Briefly, the rat was anesthetized with 1.3 g/kg *ip* urethane (Sigma Chemical, USA). The abdominal and thoracic cavities were opened and the portal vein (entry via) and the inferior vena cava (exit via) were cannulated. The livers were exsanguinated and perfused (with no recirculation) with Krebs-Henseleit bicarbonate buffer (pH 7.5), containing 1 mg/mL BSA (Sigma Chemical), at 37°C, http:/saturated with an oxygen/carbon dioxide mixture (95/5%) and at a constant flow (3–4 mL·min^−1^·g^−1^ liver). Liver viability was ensured by bile secretion and oxygen uptake monitored continuously by an oximeter (Delta OHM, Italy) connected to the efferent cannula during the experiment. The portal pressure was also continuously monitored with an open vertical column attached before the afferent cannula. After 20 min of stabilization previously determined (glucose release and portal pressure), 2 nmol AII or 40 nmol Adr (Sigma Chemical) was injected *in bolus* through the portal vein cannula. Aliquots of perfusate were collected at 0 and every 30 s until 5, 6, 8, and 10 min for glucose determination.

The agonist-induced response was observed for 5 min in the absence or presence of 17.5 μM propranolol http:/chlorhydrate (Pro; http:/Medley, Brazil) (β-AR antagonist) administrated by gavage 1 h before the experiment and/or 10 µM losartan http:/potassium (Los; EMS, Brazil) (AT1-R antagonist) and/or 25 µM prazosin http:/chlorhydrate (Pra; http:/Pfizer, USA) (α_1_-AR antagonist) both added to Krebs solution 5 min before agonist injection.

Animals were divided into 12 experimental groups (6 for AII and 6 for Adr): Control (absence of antagonists), Los, Pro, Pra, Los+Pro, and Pro+Pra. None of the antagonists *per se* altered the studied parameters.

### Liver viability – bile production and oxygen consumption

Bile was collected over approximately two periods of 10 min (before and after agonist injection) and is reported as µL·min^-1^·g^-1^ liver. As bile production was similar (0.9±0.04) in both periods, the average oxygen uptake was 2.0±0.1 µmol/g in all protocols, to confirm liver viability.

### Portal hypertensive response (PHR)

The mean values obtained for PHR 5 min before the agonist injection was considered baseline portal pressure. The difference between the pressure value observed after injection of the agonist at different times and baseline value was considered the portal pressure gain (cmH_2_O). The graph of the portal pressure gain as a function of perfusion time (min) was used to calculate the area under the curve (AUC) of portal pressure, which represents the PHR, reported as cmH_2_O/min.

### Glucose release (GluR)

The release of hepatic glucose was determined in the perfusate aliquots using the commercial kit Glucose PAP (Labtest®, Brasil). Due to the absence of glucose in the perfusion fluid, we observed continuous and linear glucose release during the stabilization period, which was considered the baseline glucose release. The difference between baseline glucose release and following agonist injection was considered the gain of glucose release, mmol·min^−1^·g^−1^ liver. The graph of the gain as a function of perfusion time (min) was used to calculate the AUC of glucose release during the experiment, represented as GluR, reported as mmol/g liver.

### Statistical analysis

Parameters were compared among groups by ANOVA and Newman-Keuls post-test with the level of significance set at P<0.05. Data are reported as means±SE. Analysis was performed using the GraphPad Prism software (version 6.0; Graph Pad Software, USA).

## Results

**Figure 1.**
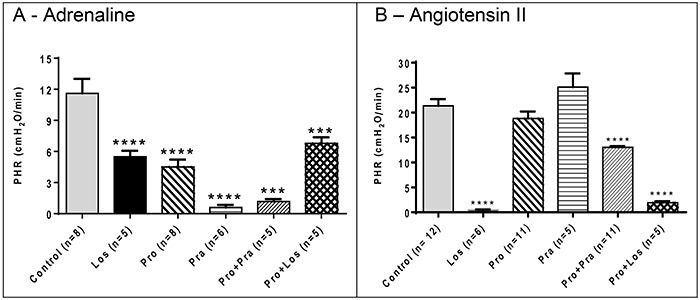
Hepatic portal hypertensive response following *in bolus* injection of 40 nmol adrenaline (Adr) or 2 nmol angiotensin II (AII). Portal hypertensive response (PHR) was calculated from the graphs “portal pressure gain × perfusion time”, after Adr or AII injection, in the presence or absence of antagonists. Los: losartan; Pra: prazosin; Pro: propranolol. Data are reported as means±SE. *P<0.05, **P<0.01, ***P<0.001, ****P<0.0001: compared with the control group; Adr: Pro *vs* Pra (**); Pro *vs* Pro+Pra (*); Pra *vs* Los (**); Pro+Pra *vs* Pro+Los (**). AII: *vs* Pra (**); Pra *vs* Pro (*) and Pra *vs* Pro+Los (***) (ANOVA followed by Newman Keuls).

**Figure 2.**
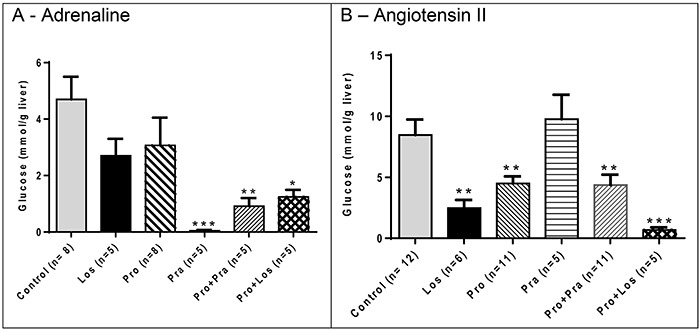
Glucose release from the perfused liver following *in bolus* injection of 40 nmol adrenaline (Adr) or 2 nmol angiotensin II (AII). Glucose release was calculated from the graphs “glucose output x perfusion time”, after Adr or AII injection, in the presence or absence of antagonists. Los: losartan; Pra: prazosin; Pro: propranolol. Data are reported as means±SE. *P<0.05, **P<0.01, ***P<0.001: compared with the control group; Adr: Pro *vs* Pra (*); AII: Los *vs* Pra (**); Pro *vs* Pra (**); Pra *vs* Pro+Pra (*); Pra *vs* Pro+ Los (***) (ANOVA followed by Newman Keuls).

Bile production (Adr: P=0.3686; AII: P=0.0829) and oxygen consumption (Adr: P=0.6302; AII: P=0.0648) were similar among groups, confirming liver viability. Both Adr and AII induced an increase in the portal pressure and glucose release. The PHR of 40 nmol Adr and 2 nmol AII were evaluated in the presence of AR and AT_1_ antagonists. Figure 1A shows that all antagonists studied decreased the PHR induced by Adr compared to the control group. Los abolished the PHR induced by AII (Figure 1B); on the other hand, AR antagonists had no effect when used alone. When antagonists were used together (Pro+Pra or Pro+Los) in the perfusion experiment, they caused a decrease in the PHR compared to the control group (P<0.0001). Pra alone and the mixture of Pro+Pra or Pro+Los decreased the glucose release induced by Adr (Figure 2A) while Los or Pro alone and the mixture of Pro+Pra or Pro+Los decreased the metabolic effect induced by AII (Figure 2B).

## Discussion

Both β-AR and AT_1-_R antagonists, as well as angiotensin-converting enzyme inhibitors are used as therapeutic drugs for several cardiac, renal, and vascular conditions, including hypertension. Although the liver is not a target organ and it might not be implicated directly in these diseases, AR and ATR are present in the cellular plasma membrane. Therefore, we studied the hepatic participation of AR and ATR in the portal hypertensive response and glucose release of Adr and AII in the rat perfused liver.

Adr is a catecholamine that interacts with hepatic AR and signals by coupling to the stimulatory G-protein Gα leading to activation of adenylyl cyclase and inducing glycogen breakdown and glucose release through cAMP-dependent pathway (12,13). Furthermore, the stimulation of AR also increases the portal pressure, and this effect is sensitive to α_1_- and β_2_-AR antagonists (14). Our results suggested that the GluR was mainly mediated by ∝_1_-AR, as reported recently by de Oliveira and co-workers (15). This high sensitivity to α_1_-adrenergic antagonists was also observed in other studies (14,15) and is strong evidence of predominant participation of α_1_-ARs in the liver (16). We also observed that the participation of β-ARs appears to have a secondary role, as it did not decrease the GluR. Los caused a significant decrease in the portal hypertensive response induced by Adr confirming the participation of AT_1_R. Although there was not a significant decrease in the GluR in the presence of Los, a partial participation of AT_1_R in this response might be important. Interestingly, when Los+Pro were added to the perfusion media, there was a sum of the effects, significantly decreasing the hemodynamic as well as the metabolic effect.

The hemodynamic effect (PHR) of AII was abolished by Los while the GluR was only diminished, as reported previously (3). The GluR induced by AII was also impaired by Pro indicating the participation of β-AR in this response. When both the ARs were blocked, the hemodynamic as well as the metabolic response was impaired showing a cumulative effect of the antagonists. Therefore, this result showed that both ARs might also be responsible for AII effects or there might be some sort of direct or indirect interaction impairing AT1-R signaling. A study with mouse cardiomyocytes showed direct interaction between β-ARs and AT_1_-Rs; this interaction would elicit a phenomenon by which selective β-AR antagonism inhibits signaling of AT_1_-receptors, whereas selective AT1-R antagonism inhibits downstream signaling of β-AR.

Moreover, the mechanism for this dual trans-inhibition of two independent receptors by a single antagonist might be via functional uncoupling of the signaling receptor from its cognate G protein (5). Therefore, whether this cross-talk between β-AR and AT1-R signaling also occurs in the hepatocytes has not yet been investigated and should be considered in the design of specific drugs. Further experiments may explain the mechanisms of this interaction and might be important in the development of drugs highly specific for pathologies involving these vasoactive peptides.
