# Erratum for: “Participation of hepatic α/β-adrenoceptors and AT_1_ receptors in glucose release and portal hypertensive response induced by adrenaline or angiotensin II” [Braz J Med Biol Res (2018) 51(12): e7526]

**DOI:** 10.1590/1414-431X20187526erratum

**Published:** 2019-01-10

L.J.T. de Araújo^1^, M.R. Nagaoka^2^, D.R. Borges^3^ and M. Kouyoumdjian^1^

^1^Departamento de Bioquímica, São Paulo SP Brasil Departamento de Bioquímica, Escola Paulista de Medicina, Universidade Federal de São Paulo, São Paulo, SP, Brasil

^2^Departamento de Biociências, Universidade Federal de São Paulo, Baixada Santista, SP, Brasil

^3^Departamento de Medicina, Escola Paulista de Medicina, Universidade Federal de São Paulo, SP, Brasil

Correspondence: M. Kouyoumdjian: <mkouyoumdjian@gmail.com>

**Erratum for:** Braz J Med Biol Res | doi: http://10.1590/1414-431X20187526

The Journal would like to correct the Author of Correspondence for the article “Participation of hepatic α/β-adrenoceptors and AT_1_ receptors in glucose release and portal hypertensive response induced by adrenaline or angiotensin II” that was published incorrectly in volume 51 no. 12 (2018) in the Brazilian Journal of Medical and Biological Research <http://dx.doi.org/10.1590/1414-431X20187526>

The correct Author of Correspondence is: M. Kouyoumdjian: <mkouyoumdjian@gmail.com>

